# Genetic diversity and variation among Akabane virus field isolates from goats in Yunnan, China

**DOI:** 10.3389/fvets.2025.1545576

**Published:** 2025-06-03

**Authors:** Jinping Wang, Aiguo Xin, Yuwen He, Jiarui Xie, Haisheng Miao, Fuxiang Li, Huafeng Gao

**Affiliations:** Yunnan Tropical and Subtropical Animal Virus Disease Laboratory, Yunnan Academy of Animal Husbandry and Veterinary Science, Kunming, China

**Keywords:** Akabane virus, goat, phylogenetic analysis, genogroup, serum antibody

## Abstract

In China, the Akabane virus (AKAVs) has been reported in several host species. However, data regarding goats is still showing a gap. Akabane virus (AKAV) is an insect-borne virus from the *Peribunyaviridae* family that in ruminant species, particularly affects pregnant animals, resulting in abortions, stillbirths, and premature broth, often with congenital abnormalities. Therefore, there is a dire need to understand the diversity of this virus in ruminants, particularly in goats. The current study aimed to investigate the genotype characteristics of goat-originated AKAVs in Yunnan, China. For this, blood samples from goats were collected for four consecutive years (2019–2023) during routine disease surveillance in Yunnan province. The serum was harvested and evaluated for the seroprevalence of the AKAVs. The seroprevalence analysis revealed that the majority of goats in Yunnan province are infected with AKAVs, with a detected prevalence of 7.69% (92/1,197 tested), even though detected seroprevalence rose as high as 27.8% in some areas. For the evolutionary analysis of AKAVs of goat, five AKAVs strains were isolated from AKAV serum-positive goat blood samples were whole genome sequenced. The phylogenetic analysis (sequenced small and medium segments) classified the AKAVs into Ia and Ib genogroups, with Ia genogroup strains being more common in Yunnan goats. However, genotype II (TJ2016 and CQ-AKAV-1-2023) emerged in China. Overall, this study emphasizes the importance of implementing prevention and control strategies for AKAVs diseases transmitted in Yunnan, China.

## 1 Introduction

Akabane disease is a viral disease caused by the Akabane virus (AKAV) species of the *Orthobunyavirus* genus of the *Peribunyaviridae* family (1, 2). It was first isolated in the Japanese village of Akabane from the mosquitoes *Aedes vexans* and *Culex tritaeniorhynchus* in 1959 and it was also a major cause of epizootics of congenital arthrogryposis and hydranencephaly in ruminants in Australia (1, 3). A wide range of wild and domesticated animals are susceptible to AKAV infection and symptomatic infections have been observed in bamboo rats, swine, sheep, and goats in recent years (4–9). In most cases, transient fever has been shown in animals without any other apparent clinical symptoms. Serum antibodies against AKAV have been detected in buffalos, camels, deer, horses, and donkeys, suggesting that more animal species can be infected by AKAV (10, 11). It affects ruminants, particularly during their pregnancy stage, and causes stillbirths, premature births with congenital malformations, and abortions. The virus is primarily transmitted by biting midges belonging to the *Culicoides* genus and by mosquitoes (12).

The virion of the *Orthobunyavirusesis* characterized by their lipid envelope and contains three segments of negative, single-stranded RNA, designated as S (small), M (medium) and L (large) which differ in sizes with approximately 0.86, 4.3, and 7 kb, respectively (3, 13). The S RNA segment encodes the nucleoprotein (N) and a non-structural protein (NSs) in overlapping reading frames. Currently, AKAVs has been classified into four genetically distinct groups (genogroups I–V), and genogroup I have been further subdivided into two subgroups (Ia and Ib) (14).

AKAV Infections have also been reported in several regions across Australia, Southeast Asia, East Asia, Africa, the Middle East, and even in Africa. Disease induced by AKAV has only been reported in bamboo rats although several AKAV strains have been isolated from cattle, mosquitoes, and goats in regular disease surveillance in China (8, 9, 15, 16). With over 14 million goats and 9 million cattle in Yunnan province, animal husbandry is a major agricultural industry in Yunnan, where ruminants are highly susceptible to AKAV infection. From 2006 to 2015, a national survey analyzed 2,731 serum samples obtained from cattle, sheep, and goats across 24 provinces in China by the serum neutralization method. The overall seroprevalence of AKAV was 21.3% in cattle (471/2,215), 12.0% (17/142) in sheep or goats, and 0% in yak (0/374) (17). Another survey carried out on 420 serum samples collected from the Yunnan province indicated that seroprevalence was 30 and 20% in cattle and goats, respectively. In the prevailing circumstances of no vaccine used against AKAV, the results indicated that AKAV infection was serious in Yunnan, China. The higher seroprevalence in tropical and subtropical Yunnan compared to the national average suggests a more common infection in this region. Therefore, this study aimed to conduct serological surveillance of AKAV infection in Yunnan province, isolate the virus, and investigate the genotype characteristics and genetic diversity of AKAV isolates in goats.

## 2 Materials and methods

### 2.1 Dual sample collection and serological testing

Serum and blood samples were collected from goats in different counties of Yunnan province between 2019 and 2023 as part of our institution’s annual surveillance program. Serum is used for antibody monitoring, and anticoagulated blood is used for virus isolation. In line with established epidemiological protocols and our routine monitoring responsibilities, systematic sampling was conducted across multiple farms in various counties to ensure a representative coverage of the target population. This approach enabled the capture of spatial and temporal variations in AKAV exposure, and the collected samples provided the basis for the subsequent serological and molecular analyses performed in this study.

The presence of AKAV antibodies was determined using a commercially available Akabane Competition ELISA Kit (ID-VET, Montpellier, France), following the manufacturer’s instructions. Serum samples that tested positive were subsequently utilized for virus isolation, as a source of antibodies in immunofluorescence assays, and for calculating the prevalence of AKAV infection.

### 2.2 Samples and virus isolation locations

**Figure 1:**
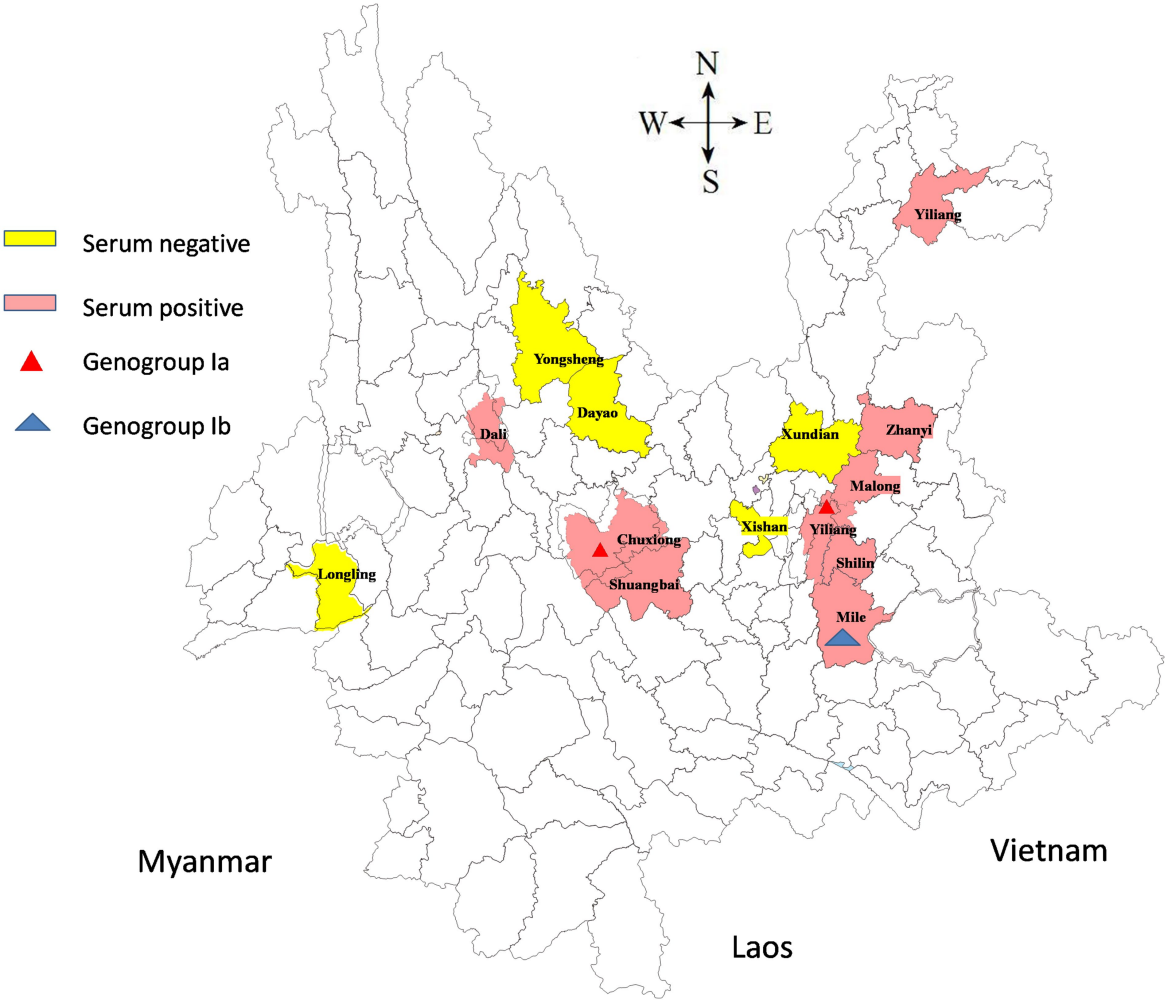
A map of Yunnan showed the geographical distribution of the sample collection sites. Serum negative areas are marked in yellow and serum positive areas in pink. The triangles indicate the counties from which the viruses were isolated in this study.

One thousand one hundred and ninety-seven serum and blood samples collected from goats in 14 counties in Yunnan between 2019 and 2023 were evaluated for the existence of AKAV antibodies. Five field isolates of AKAV used in this study are listed in Table 1. The geographical distribution of the collection locations is shown in Figure 1.

**Table 1:** Characteristics of Akabane virus isolates from goats in Yunnan province.

Strain	Location	Isolate data	Host	Genome type	Accession number
CX-01	Chuxiong city	Aug-2019	Black goat	Ia	MW194115–MW194117
YNYL-2019-AKAV	Yiliang, Kunming	Oct-2019	Sannan goat	Ia	PP918934, PP918938, PP918942
YNYL-2020-AKAV	Yiliang, Kunming	May-2020	Sannan goat	Ia	PP918935, PP918939, PP918943
YNYL-2021-AKAV	Yiliang, Kunming	Sep-2021	Sannan goat	Ia	PP918936, PP918940, PP918944
YNML-2020-AKAV	Mile, Honghe	Aug-2020	Black goat	Ib	PP918937, PP918941, PP918945

### 2.3 Virus isolation and indirect immunofluorescence assay

The baby hamster kidney cells (BHK-21) used for virus isolation were cultured in MEM supplemented with antibiotics (100 IU/mL penicillin and 10 μg/mL streptomycin) and 5% heat-inactivated foetal bovine serum (Everygreen, Hangzhou, China). Blood samples were inoculated with 1 mL of supplemented cell culture for 1 h at 37°C, then washed three times with MEM, and the flasks were incubated at 37°C in 5% CO_2_ for 3 days. The viruses were harvested and inoculated into fresh BHK-21 cells for the second passage, when cytopathic effects (CPEs) were observed. Samples without developing CPEs were considered AKAV-negative.

The BHK-21 cells were plated with the genotype Ia and Ib Akabane virus strain YNYL-2019-AKAV and YNML-2020-AKAV in 6-well plates which were inoculated with two strain isolates at a multiplicity of infection (MOI) of 0.05. Post-incubation, the cells were fixed with 4% paraformaldehyde. After three successive washes with PBS (pH 7.2), incubated with goat AKAV positive serum (diluted 1:10) for 30 min at 37°C and then stained with a 100-fold dilution of fluorescein isothiocyanate-conjugated rabbit-anti-goat antibody at 37°C for 30 min (BBI, Shanghai, China). After washing the plates with PBS, the BHK-21 cells were air-dried and examined at 200× magnification under a fluorescence microscope (Zeiss, Oberkochen, Germany). The BHK-21 cells in which the virus was not propagated were used as the negative control. Samples exhibiting specific fluorescence in the cytoplasm were identified as positive.

### 2.4 PCR amplification and sequencing

Viral nucleic acids were extracted from virus-containing cell culture supernatant using MiniBest Viral RNA/DNA Extraction Kit (TaKaRa, Dalian, China) and stored at −80°C until analysis. The PCR primers were designed based on the DHL10M110 virus strain sequences, and the amplification was performed using previously known one-step amplification methods with modifications using a GeneAMP PCR System 9700 thermocycler (Applied Biosystems, Foster City, CA). A 702-bp fragment spanning the ORF sequence of the S RNA segment was amplified, as were full-length M and L RNA segments, using four unique primer pairs as previously described (16). The amplified DNA fragments were purified using an Agarose Gel DNA Extraction Kit and cloned into the pMD-19 T cloning vector (TaKaRa, Dalian, China) according to the manufacturer’s instructions. Three positive clones were sequenced using Sanger sequencing approach. Sequences of fragments from five isolates were assembled into full-length S, M, and L genome by Vector NT1 software, respectively. The sequence of the goat AKAV isolates in Yunnan province in this study has been deposited in Genbank accession numbers shown in Table 1.

### 2.5 Sequence analysis

The sequences of five isolates open reading frame (ORF) and the deduced proteins were analyzed using DNAstar 7.0 software (DNASTAR Inc., Madison, WI, United States). For the construction of the neighbor-joining phylogenetic tree, 37 S gene segments and 36 M gene segments of AKAV’s reference genomic sequences were downloaded from GenBank. The phylogenetic trees of the coding regions in the S and M RNA segments were constructed using the neighbor-joining method in the Molecular Evolutionary Genetics Analysis program version (MEGA 7) with bootstrap values based on 1,000 replicates, the Kimura 2-parameter, and a nucleotide substitution model (18).

### 2.6 Statistical analysis

Prevalence estimates were calculated as the ratio of positive samples to the total number of samples in each group. The corresponding 95% confidence intervals were determined using the binomial proportion method with the normal approximation for groups with non-extreme proportions, and the Clopper–Pearson exact method for groups with zero positive cases. The significance of differences was assessed based on the *p*-value derived from this test, with a threshold of 0.05 considered statistically significant. All statistical analyses were performed using SAS 9.0 software.

## 3 Results

### 3.1 Virus isolation and indirect immunofluorescence assay

**Figure 2:**
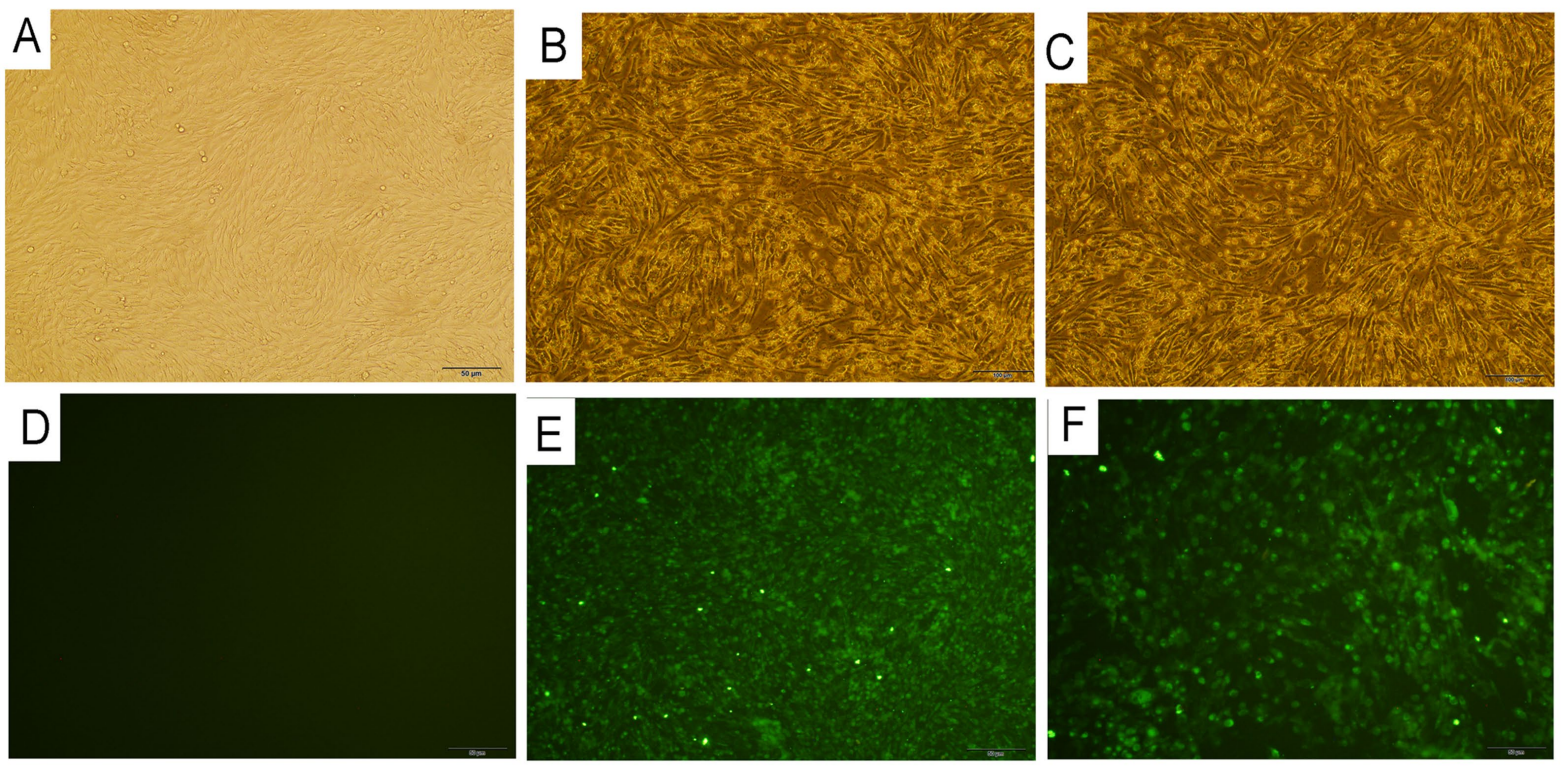
Histopathological images of BHK-21 cells infected with two different gengroup Ia and Ib Akabane virus strain YNYL-2019-AKAV and YNML-2020-AKAV. **(A)** Control BHK-21 cells. **(B)** Cytopathic effect of YNYL-2019-AKAV in BHK-21cells. **(C)** Cytopathic effect of YNML-2020-AKAV in BHK-21 cells. **(D)** Immunofluorescence results of BHK-21 cells. **(E)** Immunofluorescence results of BHK-21 cells infected with YNML-2020-AKAV after 48 h. **(F)** Immunofluorescence results of BHK-21 cells infected with YNML-2020-AKAV after 48 h.

Positive blood samples of AKAV detected by ELISA were selected for virus isolation. BHK-21 cells inoculated with the above positive blood samples indicated typical CPEs as early as 48 h. Initially, the cells began to round and nucleus condensation can be observed and 48–72 h after inoculation, most of the infected cells were detached off the culture flask. We subsequently performed three generations of cultures. All the AKAVs isolates showed similar CPEs in each passaging, signifying that the infection of the potential pathogens caused the CPEs in the inoculum (Figures 2B,C). No CPEs were observed in the he control BHK-21 cells without inoculation of the virus (Figure 2A). Five field strain of goat AKAVs were isolated and identified by specific RT-PCR and sequencing. Three fragments of the virus showed high sequence identity with AKAV, and no other viral matches were detected.

The two distinct genotype AKAV isolates were further passaged to confirm efficient propagation. Same CPEs were usually observed in BHK-21 cells infected with each isolate within 48 h post-inoculation, cells containing many nuclei were observed over time. After fixing the cells with cold acetone and staining with the positive goat serum, specific fluorescence was detected in the cytoplasm of the cells infected with YNYL-2019-AKAV and YNML-2020-AKAV (Figures 2E,F). The control BHK-21 cells without inoculation of the virus was presented in Figure 2D.

### 3.2 Comparative nucleotide and amino acid sequence analyses

The nucleotide sequences of the S, M, and L RNA segments of goat-derived AKAV isolates were determined and examined. The sequenced S RNA segments comprised a 702-nt ORF with no insertions or deletions, encoding 233 amino acid residues. The nucleotide and amino acid sequences obtained from goats in Yunnan were compared to Chinese isolates and other reference strains from other genera and genotypes. Analyzing the S gene sequences revealed that the nucleotide homology across the Yunnan isolates was 94.9–100% and 99.1–100% similar at the nucleotide and amino acid levels, respectively. When compared to other subgroups, the Yunnan strains had 94.9–100% and 96.2–100% similarity with the nucleotide sequences of genogroup Ia and Ib, respectively. Among all Yunnan strains, the YNML-2020-AKAV strain had the lowest sequence homology with Yunnan strains (94.7%) and other genogroups (96.2%) (Table 2).

**Table 2:** Comparison of the coding region segments S, M, and L (ORF) among different genogroup of AKAVs.

Comparison	Pairwise % identity (nt/aa)		
	S	M	L
Among the same farm	97.6–100 (99.1–100)	95.7–98.2 (97.1–98.8)	94.2–98.3 (98.4–99.3)
Isolates from goats in Yunnan province	94.9–100 (99.1–100)	87.8–99.6 (94.8–99.6)	90.1–99.5 (97.2–99.6)
Within China isolates	94.7–100 (98.3–100)	87.3–99.1 (93.2–99.3)	89.8–99.5 (97.2–99.6)
Among genogroup Ia	94.9–100 (98.3–100)	91.0–100 (95.1–100)	90.1–98.5 (97.2–99.6)
Among genogroup Ib	96.2–100 (99.1–100)	91.1–99.9 (97.3–99.9)	95.6/99.2
Among genogroup II	97.2–100 (99.1–100)	92.0–99.9 (96.4–99.9)	91.5–100 (97.5–100)
Genogroup III R794 to field isolates	92.0–93.7 (96.6–97.4)	84.0–86.6 (91.4–93.8)	86.2–87.8 (95.7–96.3)
Genogroup IV MP496 to field isolates	82.8–84.9 (90.6–91.5)	69.5–70.4 (73.7–75.0)	—

Open reading frame sequences of the S, M, and L RNA segments were selected to compare variation with nt—nucleotide; aa—amino acid.

The M RNA segment included a 4,266 nt ORF with no insertions or deletions, encoding 1,233 amino acid residues. The nucleotide and amino acid sequences obtained from goats in Yunnan were compared to Chinese isolates and other reference strains ranging from genogroup Ia to genogroup III R794 strain. Analysis of the M gene sequences revealed that the nucleotide homology among the Yunnan isolates was 94.9–100% and 99.1–100% similar at the nucleotide and amino acid levels, respectively. Comparisons of nucleotide (nt) and amino acid (aa) sequences among AKAV field isolates from China show that the ORF sequence of the S RNA segment was highly conserved among all the Chinese isolates (<5.1 difference in nt, <0.9 difference in aa), and for all field strains from genogroup Ia to genogroup III, the ORF sequence of the S showed the same evolutionary trend (<8.0 difference in nt and <3.4 in aa). In comparison, the L RNA ORF sequence was more variable, while the M RNA ORF sequence varied the most (Table 2).

### 3.3 Phylogenetic analyses of the S, M and partial L RNA segments

**Figure 3:**
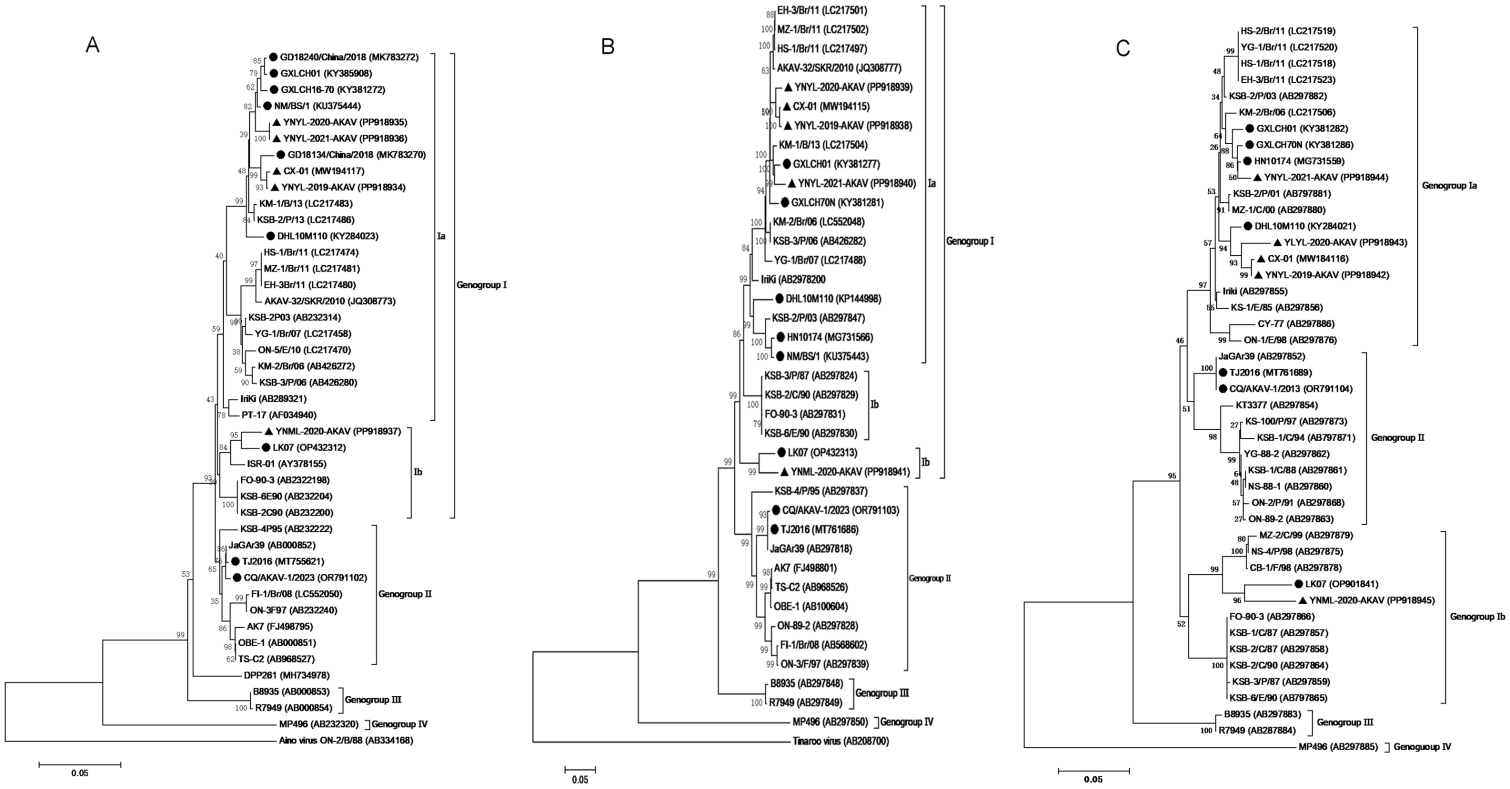
Phylogenetic analyses of the full length ORF of the small (S) **(A)**. Full length of the medium (M) RNA segment **(B)** and a partial length of the L RNA sequences **(C)**. The scale bars represent 0.05% substitution per base. Sequences obtained in this study are marked with black triangles (▲) and the previous Chinese isolates are marked with round black spots (●).

To determine the genetic links between five goat isolates from Yunnan province and other AKAV strains, we created phylogenetic trees using ORF sequences from the S and M RNA segments of 37 and 36 reference AKAV strains previously published. The S RNA segment sequences identified four goat isolate strains as belonging to genogroup Ia, along with six Chinese strains (Figure 3A). The field goat isolates of the YNML-2020-AKAV strain were assigned to genogroup Ib, along with another Chinese bovine sample, LK07. Phylogenetic tree based on M RNA segments revealed that four goat isolate strains belonged to genogroup Ia, grouping with five additional Chinese strains from various hosts. Genogroup Ib includes the Yunnan goat isolate YNML-2020-AKAV and the inner Mongolian bovine isolate LK07. Since 2004, all genogroup Ia strains reported in China have shown evidence of evolutionary divergence. Local mosquito isolate DHL10M110 is genetically closer to Korea isolate KSB-2P/13 than Yunnan small ruminant isolate (Figure 3B). The partial L RNA segment corresponding to the 5′ end of the cDNA of 45 AKAV isolates which contains 559 nt in length and potentially encoded 181 aa were compared with the reference strains. The sequence alignment results indicate that L RNA segment of YNML-2020-AKAV has the maximum differentiation among strains in Yunnan (Figure 3C). Three isolates from the same goat farmer in 3 years suggest that virus recombination has occurred. This genomic data indicates that distinct sub-genogroup and genogroup AKAVs proliferated in different parts of China in the past.

### 3.4 Amino acid analysis of S and NS protein of the Chinese isolates of AKAVs

The amino acid sequences of nucleoprotein S and non-structural protein NS of 14 Chinese strains of AKAVs were compared with the amino acid sequences of reference strain Iriki (Ia). The results demonstrated that there were no deletion or frame shift mutations in nucleotide and amino acid formation of all genotype I and genotype II strains. The amino acid locations of S protein in Chinese isolates showed little alterations. All genogroup I and II strains were made up of 233 amino acids encoded by 699 nucleotides. The YNML-2020-AKAV isolate has only one amino acid change, 206 (S → N). The strains with the highest mutation frequency were CX-01, YNYL-2019-AKAV, YNYL-2020-AKAV, and YNYL-2021-AKAV. They all contained three mutation sites, with the common locus of variation being 115 (E → S). The remaining nine Chinese isolates were likewise susceptible to change at these loci (Table 3). The YNML-2020 isolate only exhibited one amino acid change, 206 (S → N). The non-structural protein NS is made of 91 peptides encoded by 273 nucleotides. Compared with the S protein, the non-structural protein is more prone to change. The Yunnan goat isolates YNYL-2019-AKAV and YNYL-2020-AKAV both have five mutation sites, namely 15 (G → D), 73 (I → T), 75 (Q → R), 76 (I → T), and 88 (S → F). YNML-2020-AKAV (Ib), which had two sites of variation, 22 (S → N), and one common site 73 (I → T), the gene LK07 (IB) was the lowest variation, with only one mutation in nucleoprotein S and non-structural protein NS. In addition, CQ/Akav-1/2023 (II) non-structural proteins only produce one amino acid change at position 73 (I → T). The analysis of amino acid variation based on S and NS showed that the classification of genotype and sub-genotype had low similarity with the variation, and the accumulation of nucleotide variation did not produce the corresponding amino acid variation, this mutation is more of a nonsense mutation.

**Table 3:** Amino acid changes in the deduced sequences of the N and NS protein of the Chinese AKAV isolates compared to the reference strain.

Strains	N protein						NS protein									
Iriki (Ia)	22A	104V	115E	202K	206S	221A	14Q	15G	22S	33E	70F	72S	73I	75Q	76I	88S
CX-01	—	I	L	—	N	—	R		—	G	—	—	T	—	T	—
YNYL-2021	—	—	L	R	N	—	—	D	—	—	—	—	T	R	T	F
YNYL-2020	—	—	L	R	N	—	—	D	—	—	—	—	T	R	T	F
YNYL-2019	—	I	L	—	N	—	R	—	—	—	—	—	T	—	T	—
DHL10M110	—	—	L	—	N	—	—	—	N	—	—	—	T	—	T	—
GD18240/China/2018	—	—	L	—	N	—	—	—	—	—	—	—	T	—	T	F
GXLCH01	—	—	L	—	N	—	—	—	—	—	—	N	T	—	T	F
GD18134/China/2018	—	I	L	—	N	—	—	—	—	—	—	—	T	—	T	—
GXLCH16-70	—	—	L	—	N	—	—	—	—	—	—	—	T	—	T	F
NM/BS/1	—	—	L	—	N	T	—	—	—	—	—	—	T	—	T	F
YNML-2020	—	—	—	—	N	—	—	—	N	—	—	—	T	—	—	—
Lk07	—	—	—	—	T	—	—	—	—	—	—	—	T	—	—	—
TJ2016	—	—	—	—	G	—	—	—	—	—	S	—	T	—	—	—
CQ/AKAV-1/2023	22V	—	F	—	G	—	—	—	—	—		—	T	—	—	—

### 3.5 Statistical analysis of AKAV infection

In this study, 1,197 serum samples collected from goats in Yunnan between 2019 and 2023 were evaluated for the existence of AKAV antibodies. There was evidence of AKAV antibodies in Yunnan goats from 14 different counties. Overall, AKAV seroprevalence in goats was 7.69% (92/1197 tests) (Table 4). From 2019 to 2023, approximately 4.2–5.5% of goat samples tested positive for AKAV using ELISA. The positive rate for AKAV samples from goats was high in some regions of Yunnan, implying that AKAV is still a significant pathogen in Yunnan.

**Table 4:** Prevalence of AKAV antibodies in goat sera collected from Yunnan, 2019–2023.

Location (county)	Data collected	Total samples	Number positive	Prevalence (%)
Shilin, Kunming	Sep-19	92	6	6.5
Chuxiong city	Aug-19	22	5	22.7
Dayao, Chuxiong	May-20	100	0	0
Xishan, Kunming	Jul-22	35	0	0
Dayao, Chuxiong	Aug-22	92	0	0
Malong, Qujing	May-22	54	15	27.8
Yiliang, Zhaotong	Jun-22	23	2	8.7
Yiliang, Zhaotong	May-21	84	20	23.8
Xundian, Kunming	Jul-22	32	0	0
Yiliang, Kunming	Feb-22	20	0	0
Yiliang, Kunming	Oct-22	72	6	8.3
Yiliang, Kunming	Nov-22	70	4	5.7
Mile, Honghe	Aug-22	37	7	18.9
Dali city	Nov-22	40	5	12.5
Longling, Baoshan	Feb-22	40	0	0
Yongsheng, Lijiang	Jul-23	96	0	0
Mile, Honghe	May-23	65	7	18.9
Yiliang, Kunming	Jun-23	103	8	6.3
Shuangbai, Chuxiong	May-23	96	6	3.1
Zhangyi, Qujing	Oct-23	24	1	4.2

Prevalence characteristics of AKAV in goats in part of the Yunnan province, China.

Based on 809 serum samples data from nine positive regions, a chi-square test comparing the prevalence between 2019 and 2021 revealed a statistically significant difference (*p* ≈ 0.007, <0.05). The statistical significance of differences in AKAV seroprevalence between the lowest Shuangbai county and the highest Malong county was determined by using the *χ*^2^ test (*χ*^2^ = 10.45, *p* < 0.01), demonstrating an extremely significant difference in AKAV infection in different county. Significance differences in AKAV seroprevalence between May and August was determined by using the *χ*^2^ test (*χ*^2^ = 9.61, *p* < 0.01). Difference between May and September was determined by using the *χ*^2^ test (*χ*^2^ = 0.06, *p* = 0.08). There was no difference in infection rates between the 2 months. Statistical analysis based on time shows AKAV infection rate is closely related to time.

## 4 Discussion

AKAV infections have been widely reported in Australia and various Asian countries, ranging from tropical Indonesia to temperate countries like Japan and Korea. The prototype strain of AKAV, JaGAr39, was first isolated in Japan from mosquitoes in 1959 (19). Since then, the disease has been detected in other countries as well. AKAV was first isolated in China in 1998 from mosquitoes collected during a disease outbreak in Shanghai (20). This followed reports of the Akabane disease was widely distributed in Guangdong, Hunan, Shandong, Guangxi, and Yunnan. The first AKAV was isolated in Yunnan province in 2004 from mosquitoes (21). Subsequently, viruses have been successfully isolated from cattle and goats. Genogroup Ia is prevalent in southern China but sporadically appears in Japan and South Korea (8, 9, 14, 22–26). Genogroup Ib has been detected sporadically in Israel, Turkey, and Indonesia (6, 25, 27–29). Furthermore, genogroup II is endemic to Japan and South Korea (22, 25, 30), whereas genogroup III only exists in Australia, and genogroup IV is found only in Africa (25, 31). In this study, we collected positive blood samples from surveyed goat farms for virus isolation and further genetic characteristics analysis. Analysis based on the S and M segments indicated that AKAV isolates clustered into two clades corresponding to genogroup Ia and Ib. Previous studies indicated that genogroup Ia was the predominant subgroup in Yunnan and adjacent provinces (8, 9, 16, 23). However, our results showed the presence of genogroup Ib in Yunnan. Additionally, recent analyses of Chinese epidemic strains revealed that genogroup II also appears in regions with different geographical and climatic conditions, such as subtropical Chongqing and temperate Tianjin (32). The emergence of different subgroups may suggest potential virus recombination and changes of the virulence reassortment experiments of the Korean isolates indicate that the S segment, especially the NS protein, is associated with the pathogenicity (33). It is unclear whether the observed differences in pathogenicity are due to the emergence of different genogroup isolates originating from China or if further experimental studies are required to confirm this association. We isolated viruses from blood samples collected from the same monitoring site over 3 years from 2019 to 2021. The nucleotide homology of the S gene among the three isolates, YNYL-2019-AKAV, YNYL-2020-AKAV, and YNYL-2021-AKAV, was 97.6 to 100%. YNYL-2020-AKAV and YNYL-2021-AKAV shared identical nucleotide sequences. The nucleotide homology of the M and L genes ranged from 95.7 to 98% and 94.2 to 98.3%, respectively, with significant variations observed in the 2019 strain compared to the strains from the following 2 years. The source of these variations remains unclear, whether they resulted from point mutations or different mosquito vectors. There are currently four species of mosquitoes in China, namely *A. vagas, C. tritaeniorhynchus*, *A. sinensis*, and *C. quinquefasciatus*, which have been confirmed to be able to transmit the virus (21, 23). Genetic analysis of all five Yunnan goat strains revealed that the nucleotide homology of the S gene ranged from 94.9 to 100%, YNML-2020-AKAV had the lowest nucleotide and amino acid homology with all strains, with the greatest difference observed with YNYL-2019-AKAV, with only 94.9% homology. The accumulated mutations formed a new subgroup, genogroup Ib. Analysis of the NS gene indicated that non-structural protein variation is more frequent than S protein variation. The Yunnan goat isolates YNYL-2019-AKAV and YNYL-2020-AKAV both had five variable sites: 15 (G-D), 73 (I-T), 75 (Q-R), 76 (I-T), and 88 (S-F). The YNML-2020-AKAV (Ib) isolates had two variable sites, 22 (S-N) and 73 (I-T). The mutation in the NS region may indicate differences in host antiviral defense and contribute to the regulation of host protein synthesis and apoptosis (34). However, further reverse genetic investigations are needed to assess whether these functions correlate with NS and its variations. Strengthening vector classification and virus-carrying studies in more farms will help us better understand the transmission of this disease in Yunnan.

Previous serological monitoring indicated that, regardless of the humid and hot south Hainan or the dry and cold north Inner Mongolia, ruminants in most of the monitored provinces, even in the Qinghai-Tibet Plateau, tested positive for AKAV (17). Serologic surveillance has shown that AKAV seroprevalence in cattle occurred in the southeast of China, in Guangdong province in 2011 (56.6%) and Hainan province in 2013 (50.0%) and the results also indicate that AKAV is also distributed in higher altitude region and high latitude regions such as Haibei, Qinghai province in 2012 (8.1%) and Changchun, Jilin province in 2013 (20.8%) (17). Our study of goat serological monitoring in different regions of Yunnan province from 2019 to 2023 showed that the overall AKAV seroprevalence in goats was 7.7% (92/1,197 tested). However, the monitoring results revealed inconsistent infection rates among different geographical locations. Even in areas geographically close to each other, some farms exhibited high positivity rates while others nearby remained uninfected. Positive rate surveys indicated no significant difference between the lowest altitude area, Mile city at 1350 meters, and the highest altitude area, Malong county at 2,150 meters. Additionally, areas with higher altitudes and latitudes also showed positive infections, indicating AKAV is widely distributed in Yunnan province. Although seroprevalence of AKAV was observed at higher altitudes, vector surveillance was not conducted in this study. So far, we cannot confirm the presence of vectors in these areas, and other factors such as animal movement, shipment, or alternate transmission routes may have contributed to the exposure.

Classical clinical symptoms of AKAV infection mainly include reproductive failures, such as abortion, stillbirth, premature birth, and congenital deformities known as arthrogryposis-hydranencephaly syndrome (14). Although high seroprevalence has been detected in China, the disease usually presents as asymptomatic or mild, with only transient fever and mild neurological disorder during the rainy season when the proliferation of biting midges and mosquitoes peak. In Yunnan, the tropical to subtropical monsoonal climate and varied terrain are conducive to the proliferation of vectors, including biting midges and mosquitoes. The continued detection of AKAV antibodies in ruminants, coupled with the isolation of distinct viral genogroups, emphasizes the need for systematic studies on vector ecology and pathogen transmission. Such investigations are essential for understanding the transboundary movement of AKAV, particularly in light of increasing commercial and animal trade activities that may facilitate viral spread across borders. Systematic studies on AKAV epidemiology in this region are needed, requiring more extensive monitoring and collaborative research.

## 5 Conclusion

This study presents a molecular characterization of goat AKAV isolates from Yunnan province. The data given in this study indicates that genogroup Ia and Ib exist in Yunnan. Furthermore, genogroup II AKAVs appeared in many regions with vastly varied climate types, yet the dominant AKAV field strain in Yunnan and across the country remained genogroup Ia. We also completed seroprevalence surveys in parts of Yunnan, and the results indicate that the infection is serious. Given the prevalence rate in ruminants, this viral pathogen remains a continuing hazard to livestock in Yunnan.

## Data Availability

The datasets presented in this study can be found in online repositories. The names of the repository/repositories and accession number(s) can be found in the article/supplementary material.
